# Supplementary material for: Superhigh moduli and tension-induced phase transition of monolayer gamma-boron at finite temperatures
Source: Sci Rep. 2016 Mar 16;6:23233. doi: 10.1038/srep23233 (PMC4793268; doi:10.1038/srep23233)
Supplement: Supplementary Information [file srep23233-s1.pdf]

Superhigh moduli and tension-induced phase transition of monolayer gamma-boron at finite temperatures

Junhua Zhao[1]*, Zhaoyao Yang[1], Ning Wei[2], Liangzhi Kou[3]*

[1] *Jiangsu Key Laboratory of Advanced Manufacturing Equipment and Technology of Food, Jiangnan University, 214122, Wuxi, China*

[2] *College of Water Resources and Architectural Engineering, Northwest A&F University, Yangling 712100, China*

[3] *School of Chemistry, Physics and Mechanical Engineering Faculty, Queensland University of Technology, Garden Point Campus, QLD 4001, Brisbane, Australia*

Each initial structure is optimized and then is run for 500 ps at different temperature until the pressure and energy of the system are stable, respectively, keeping both the given temperature and the pressure P=1 atm (the time step Δt=0.1 fs), in the NPT ensemble controlled by the Nose-Hoovers thermostat[1,2]. To obtain the mechanical properties, the above equilibrium structure from NPT ensemble is kept in NVT ensemble for 200 ps and the mechanical properties under uniaxial and biaxial tension as well as shear deformation are derived. To obtain the reliable MD results and save the computational cost, we set the strain rate at 1×10^{-5}/fs under uniaxial tension and shear deformation, in which such strain rate has been also validated for other materials (such as BP, carbon nanotubes and MoS_2[3-5]). Periodic boundary

*Corresponding author. *Email address*: (JZ) junhua.zhao@163.com, (LK) liangzhi.kou@qut.edu.au

conditions are applied in all three directions. Due to the limitation of the two-dimensional (2D) calculations in LAMMPS, the wrinkle phenomenon can not be observed in pure 2D calculations. Therefore, the thickness of the vacuum layers (equal to 40 Å) are added on the monolayer γ-B_{28} and under the monolayer γ-B_{28}, respectively, which will avoid the effect of the periodic boundary condition on their mechanical behaviors.

First-principles calculations based on the DFT were carried out using the Vienna *Ab Initio* Simulation Package[6]. The exchange correlation interaction was treated within the generalized gradient approximation (GGA) in the form proposed by Perdew, Burke, and Ernzerhof (PBE)[7]. The atomic positions were relaxed until the maximum force on each atom was less than 0.01 eV $Å^{-1}$. The energy cutoff of the plane waves was set to 400 eV with an energy precision of 10^{-5} eV. For the 2D structures, the Brillouin zone was sampled by using a 10 × 8 × 1 Monkhorst-Pack grid. The lattice constants of bulk γ-B_{28} are used for the monolayer simulation, which are 5.62 and 6.987 Å along zigzag and armchair direction respectively. The vacuum space was set to at least 10 Å along thickness direction in the calculations to minimize artificial interactions between the neighbouring slabs.

Fig. S1 shows the Young's moduli, ultimate stresses and ultimate strains at different temperatures under uniaxial tension along the zigzag and armchair directions, respectively. The Young's moduli and ultimate stresses decrease with increasing temperature (from 1 K to 400 K) along the zigzag direction, while they are insensitive with the temperatures along the armchair directions. Moreover, the ultimate strains

along both zigzag and armchair directions decrease with increasing temperature. The mechanical property shows strongly anisotropic at finite temperatures, while the Young's modulus is close with each other at T= 300 K along the zigzag and armchair directions.

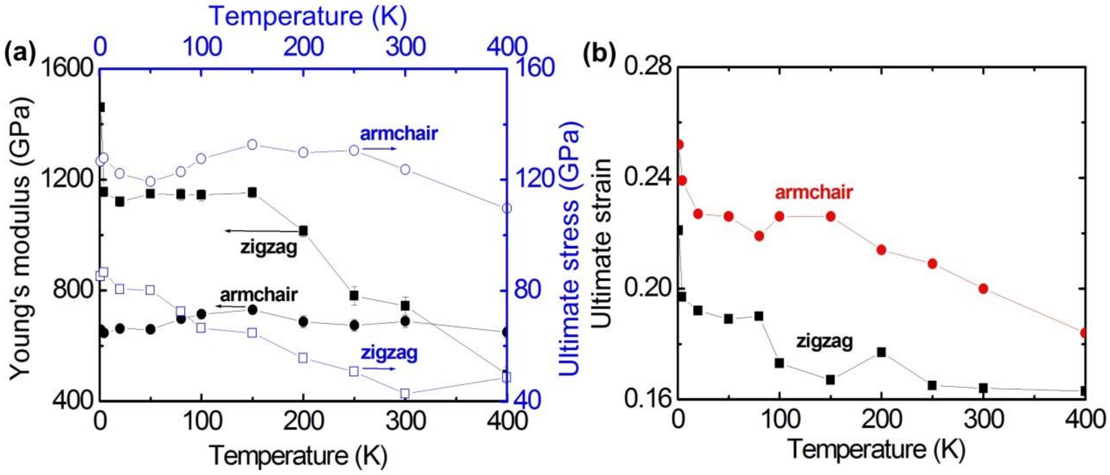

Fig. S1 (a) The Young's moduli and ultimate stresses for different temperatures under uniaxial tension; (b) The ultimate strains for different temperatures under uniaxial tension.

Fig. S2 shows the stress-strain curves of the monolayer γ-B_{28} under shear along the zigzag and armchair directions at temperature T= 4.2 K and T= 300 K, respectively. The shear moduli along the zigzag direction are 281±6 GPa at T= 4.2 K and 264±10 GPa at T= 300 K, while they along the armchair direction are 303±7 GPa at T= 4.2 K and 301±11 GPa at T= 300 K, respectively. The sawtooth-shape phenomenon can be observed in all curves, as shown in Fig. S2a. The wrinkling behavior is the main reason that leads to the sawtooth-shape phenomenon. Unlike a monolayer BP and a graphene sheet, the wrinkling behavior of monolayer γ-B_{28} at T= 300 K can be more easily observed than that at T= 4.2 K after the ultimate shear stresses, while the

wrinkles of a monolayer BP and a graphene sheet appear before the ultimate shear stresses. The main reason is probably caused by the superhigh modulus and the high thickness of monolayer γ-B_{28}. Since the wrinkles can lead to the softening of the material, the role of wrinkles is significant in two-dimensional materials[4]. To understand the effect of the wrinkles on the mechanical behavior of monolayer γ-B_{28}, we study the growth of wrinkles (the amplitude ω and wavelength λ) under shear deformation. The ratio of the amplitude to the wavelength of wrinkles at 4.2 K can be calculated directly from the MD results. The ratio from the available theory[8] can be expressed as

$$\frac{\omega}{(\lambda/2)} = \frac{\sqrt{2(1-v)\gamma}}{\pi}, \tag{1}$$

where ω is the amplitude, λ is the wavelength, v is the Poisson's ratio, and γ is the shear strain. The Poisson's ratios are chosen as 0.11[9] and 0.38 (from present MD results in NPT ensemble). The comparison between MD and theory is shown in Fig. S2. The present MD results agree well with those from the theory.

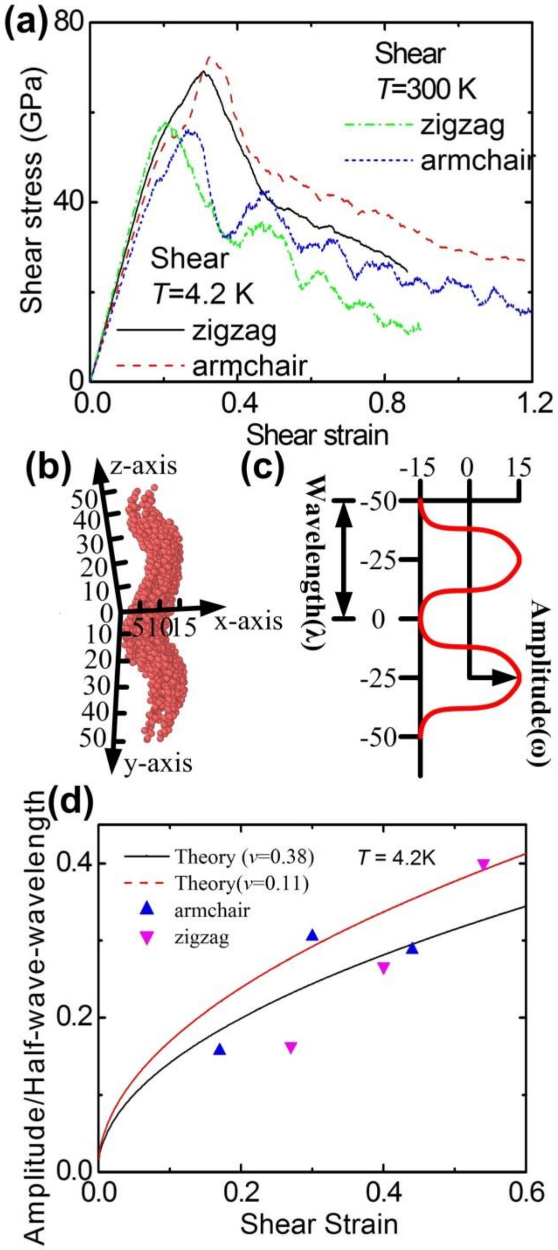

Fig. S2 The stress-strain curves for various temperatures under shear deformation and the wrinkle structures in monolayer γ-B_{28}. (a) The stress-strain curves for various temperatures along the zigzag and armchair direction under shear deformation. (b) The wrinkles in armchair monolayer γ-B_{28} sheet at 0.30 strain. (c) The schematic illustration of wrinkles. (d) Ratio of amplitude to half-wavelength vs shear strain at different temperatures comparison with the theoretical method.

The other key issue is whether the new phase can be kept stable at different temperatures along the zigzag direction after unloading process in Fig. 3a. Fig. S3 shows the final structures along the zigzag direction after unloading process from strain ε=15% at different temperatures from 1 K to 300 K, in which the new phase can be kept well after unloading process.

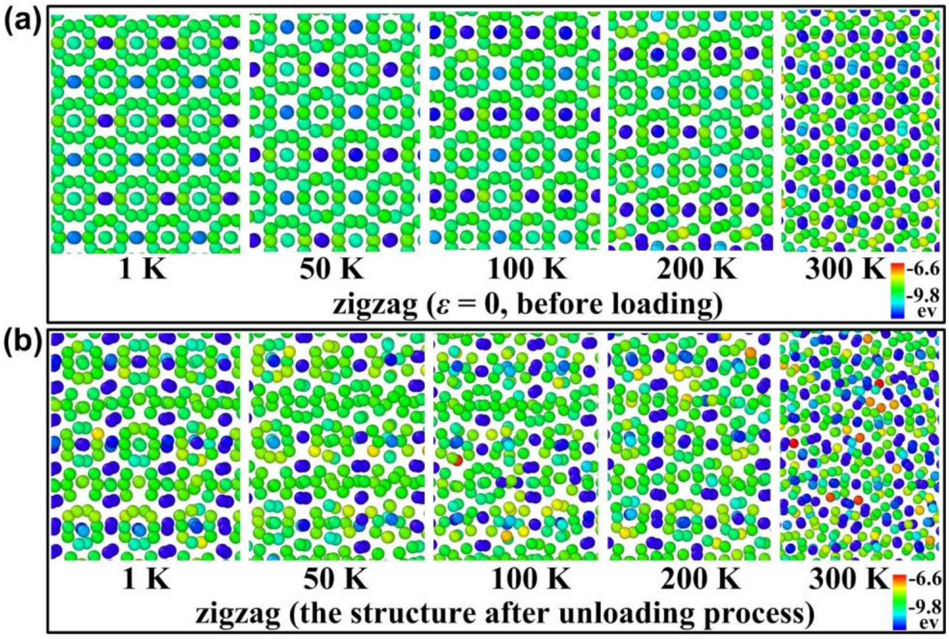

Fig. S3 The initial structures (strain ε=0, before loading), the unloading structures (after unloading process from strain ε=15%) and the potential energy per atom at different temperatures for the monolayer γ-B_{28} along the zigzag direction. (a) Initial structures (strain ε=0, before loading); (b) Unloading structures (after unloading process from strain ε=15%).

References:

[1] Nose, S. A. A unified formulation of the constant temperature molecular dynamics methods. *J. Chem. Phys.* **81**, 511-519 (1984).

[2] Hoover, W. G. Canonical dynamics: equilibrium phase-space distributions. *Phys.*

Rev. A **31**, 1695-1697 (1985).

[3] Zhao, J., Kou, L., Jiang, J. W. & Rabczuk, T. Tension-induced phase transition of single-layer molybdenum disulphide (MoS2) at low temperatures. *Nanotechnology* **25**, 295701 (2014).

[4] Yang, Z., Zhao, J. & Wei, N. Temperature-dependent mechanical properties of monolayer black phosphorus by molecular dynamics simulations. *Appl. Phys. Lett.* **107**, 023107 (2015).

[5] Wu, J., Nagao, S., He, J. Y. & Zhang, Z. L. Carbon Nanotubes: Nanohinge-Induced Plasticity of Helical Carbon Nanotubes. *Small* **9**, 3561-3566 (2013).

[6] Kresse, G. & Furthmüller, J. Efficient iterative schemes for ab initio total-energy calculations using a plane-wave basis set. *Phys. Rev. B* **54**,11169-11186 (1996).

[7] Perdew, J. P., Burke, K. & Ernzerhof, M. Generalized gradient approximation made simple. *Phys. Rev. Lett.* **77**, 3865-3868 (1996).

[8] Wong, Y. W. & Pellegrino, S. Wrinkled membranes Part I. Experiments. *J. Mech. Mater. Struct.* **1**, 3-24 (2006).

[9] Zhou, W., Sun, H. & Chen, C. Soft bond-deformation paths in superhard gamma-boron. *Phys. Rev. Lett.* **105**, 215503 (2010).
